# Optical Microscopy Using the Faraday Effect Reveals *in Situ* Magnetization Dynamics of Magnetic Nanoparticles in Biological Samples

**DOI:** 10.1021/acsnano.3c08955

**Published:** 2024-02-05

**Authors:** Maneea Eizadi Sharifabad, Rémy Soucaille, Xuyiling Wang, Michael Rotherham, Tom Loughran, James Everett, David Cabrera, Ying Yang, Robert Hicken, Neil Telling

**Affiliations:** † School of Pharmacy and Bioengineering, Keele University, Guy Hilton Research Centre, Thornburrow Drive, Stoke-on-Trent ST4 7QB, United Kingdom; ‡ Department of Physics and Astronomy, University of Exeter, Stocker Road, Exeter EX4 4QL, United Kingdom; § Healthcare Technologies Institute, School of Chemical Engineering, University of Birmingham, Heritage Building, Mindelsohn Way, Edgbaston, Birmingham B15 2TH, United Kingdom

**Keywords:** magnetic hyperthermia, magneto-optics, magnetic nanoparticles, fluorescence microscopy, nanoscale biomaterials, nanoscale biomineralization

## Abstract

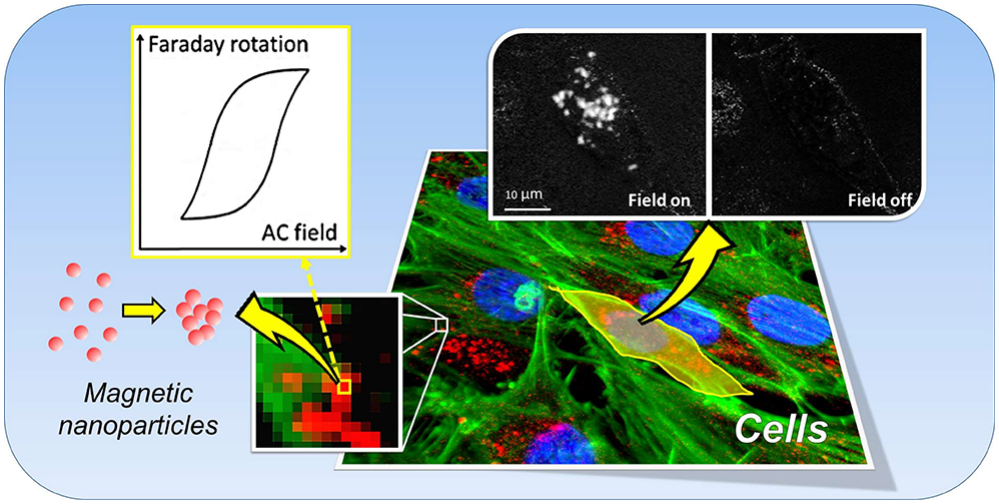

The study of exogenous and endogenous nanoscale magnetic material in biology is important for developing biomedical nanotechnology as well as for understanding fundamental biological processes such as iron metabolism and biomineralization. Here, we exploit the magneto-optical Faraday effect to probe intracellular magnetic properties and perform magnetic imaging, revealing the location-specific magnetization dynamics of exogenous magnetic nanoparticles within cells. The opportunities enabled by this method are shown in the context of magnetic hyperthermia; an effect where local heating is generated in magnetic nanoparticles exposed to high-frequency AC magnetic fields. Magnetic hyperthermia has the potential to be used as a cellular-level thermotherapy for cancer, as well as for other biomedical applications that target heat-sensitive cellular function. However, previous experiments have suggested that the cellular environment modifies the magnetization dynamics of nanoparticles, thus dramatically altering their heating efficiency. By combining magneto-optical and fluorescence measurements, we demonstrate a form of biological microscopy that we used here to study the magnetization dynamics of nanoparticles *in situ*, in both histological samples and living cancer cells. Correlative magnetic and fluorescence imaging identified aggregated magnetic nanoparticles colocalized with cellular lysosomes. Nanoparticles aggregated within these lysosomes displayed reduced AC magnetic coercivity compared to the same particles measured in an aqueous suspension or aggregated in other areas of the cells. Such measurements reveal the power of this approach, enabling investigations of how cellular location, nanoparticle aggregation, and interparticle magnetic interactions affect the magnetization dynamics and consequently the heating response of nanoparticles in the biological milieu.

Nanoscale magnetic materials, observed in biological systems ranging from simple microbes to the human brain, typically consist of iron oxide biominerals, although metallic species have also been observed.^1−4^ In this context, their presence has led to questions regarding how their chemical and magnetic properties could underlie both normal biochemical functions as well as the pathology of diseases.^5,6^ However, the biocompatibility and useful magnetic properties of some iron oxide minerals, such as (surface-oxidized) magnetite, have driven the synthesis and application of nanoparticles of these materials, which can be introduced into biological systems for a host of biomedical applications. Such applications exploit the sensitivity of the magnetic nanoparticles (MNPs) to respond to remote magnetic fields, enabling them to act as local mediators of physical properties, such as force and heat. Varying types and sizes of MNPs, used in combination with suitable DC or AC, homo or heterogeneous magnetic fields, have been explored for a wide range of applications in biomedicine, including their use as therapeutic and regenerative medicine tools.^7−9^ For example, the use of MNPs and gradient magnetic fields can serve to induce forces that can be used to shuttle MNP-drug delivery vehicles to target sites^10,11^ or to stimulate mechanosensitive cell signaling and manipulate processes such as cell migration and growth.^12−16^

In addition to magnetic forces, the ability of MNPs to convert electromagnetic energy to heat under high frequency (50–1000 kHz) AC magnetic field stimulation has led to a surge of interest in using this magneto-thermal effect to control heat-sensitive cellular responses. For example, recent studies have shown that MNP induced heating can be used to remotely control processes such as adrenal hormone stimulation,^17^ or the differentiation of cancer cells.^18^ However, the most widely studied application to date is magnetic hyperthermia for cancer therapy. This technique utilizes the sensitivity of cancer cells to elevated temperature, enabling tumor growth to be slowed or stopped by transient heating to 40–46 °C for periods of 30 min or more, while also increasing tumor sensitivity to chemotherapy and radiotherapy.^19,20^ A crucial advantage of using MNPs for heating (magnetic hyperthermia) is that local heating can be generated specifically at the location of the MNPs.^21^ Hence magnetic hyperthermia offers a method for truly local and targeted thermotherapy, on a scale commensurate with individual cancer cells and without causing damage to surrounding healthy tissue, making it extremely attractive as an anticancer treatment.

Previous clinical trials using magnetic hyperthermia have relied on injecting concentrated MNP fluids directly into a tumor in order to obtain the desired local heating response.^22,23^ However, this approach excludes the possibility of targeting individual cancer cells, which is essential to effectively treat the whole cancer, including metastases. For cellular based hyperthermia, MNPs must be prepared which show strong heating effects even at dilute concentrations and under the modest AC field conditions applicable to clinical settings. Many promising MNP materials have been developed by numerous groups in this area.^24−29^ However, it has been found that magnetic hyperthermia is altered following MNP association with cells, due to the modification of their AC magnetic properties caused by effects such as interparticle interactions.^25,30−33^

To assess and optimize the biological heating performance of MNPs, it is therefore necessary to measure their AC magnetic response *in situ*. At a given AC field frequency, the heating power of the particles (referred to as the Specific Absorption Rate) scales directly with the area of the AC hysteresis loop measured from the particles.^34,35^ Previous work in this area by a number of groups, including our own, have shown that coil based AC magnetometers are effective for determining the average AC hysteresis response from a population of cells associated with MNPs.^32,36,37^ However, these methods are limited in their extent to help understand how different structural arrangements of MNPs affect their magnetization dynamics and consequently AC hysteresis response. This knowledge is essential for biological applications where nanoparticles can take many different aggregated forms. Computational studies on the effect of interparticle magnetic interactions in different nanoparticle arrangements, have demonstrated dramatic changes to the AC hysteresis response,^38−40^ with consequent impacts expected for their heating performance. However, confirmational experiments are usually confined to population-averaged approaches which cannot differentiate between the different aggregate types that could occur in biological systems. Such information is vital in order to develop MNPs that can be designed to take advantage of cellular location as well as to understand how to tune AC field conditions to compensate for changes in the AC magnetic response of the MNPs in cellular milieu.

By exploiting the well-known Faraday effect, we present here a proof-of-concept method that we use to map the distribution of MNPs in cells via their AC magneto-optical susceptibility signal and to measure their intracellular AC hysteresis response at submicron resolution. This method is combined with the use of frequency-domain fluorescence techniques, which we use to identify specific biological structures via cytochemical staining. This combined modality also offers potential additional functionality such as the ability to probe local environmental conditions and cell responses *in situ*. We thus demonstrate a powerful form of magneto-optical biological microscopy that is ideally suited to study the subcellular magnetic behavior that defines biological magnetic hyperthermia. Beyond this, the method will have broader applications where magnetic imaging and local AC magnetometry are required at submicrometer length scales.

## Results and Discussion

### Operation of the Microscope

**Figure 1:**
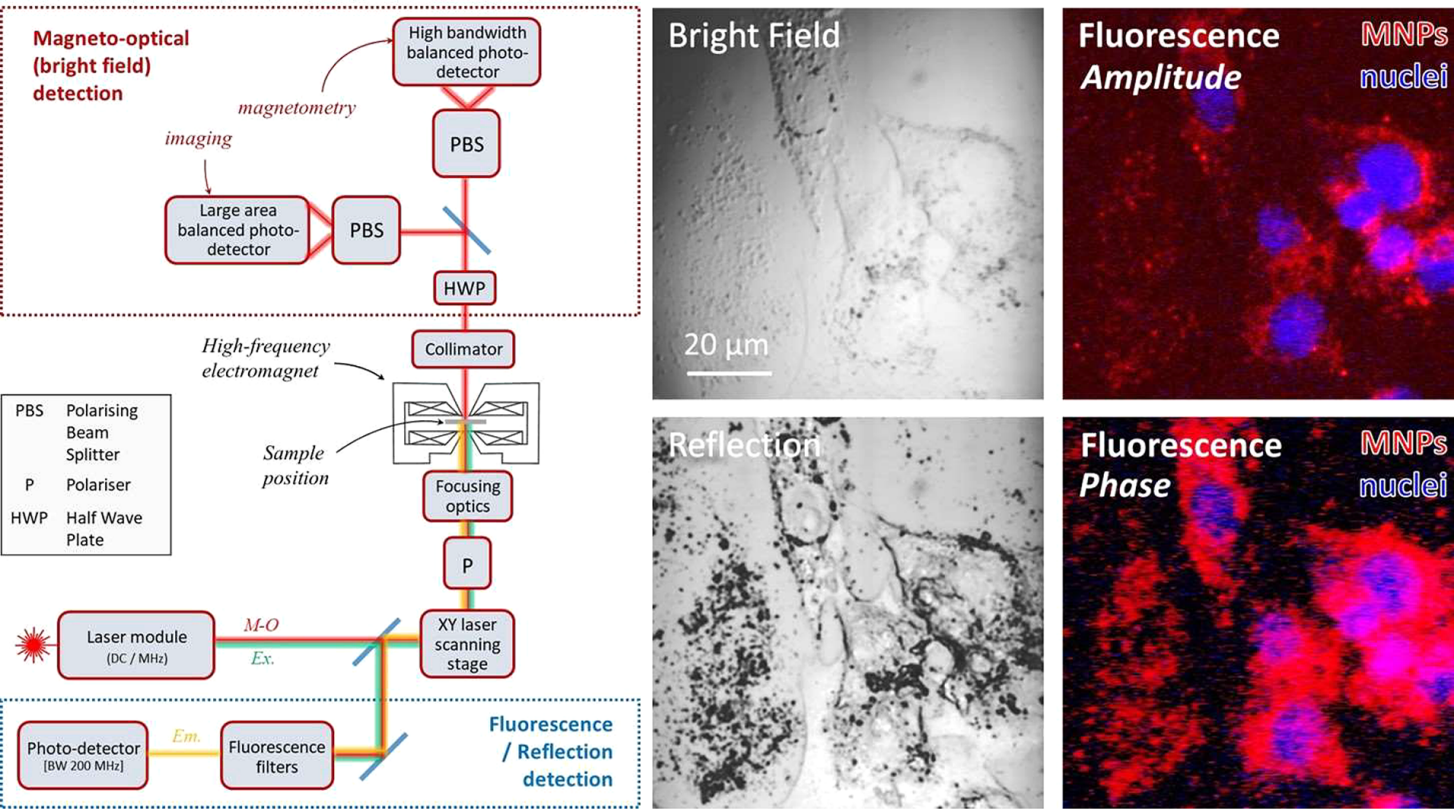
Design and optical modes of the microscope. Schematic (left) showing the layout of the combined magneto-optical and fluorescence microscope, indicating also the optical pathway for magneto-optical (M-O) imaging and fluorescence excitation (Ex.) and emission (Em.). Representative images (right) obtained from fixed osteosarcoma cells (MG63 cell line) following their incubation with fluorescently tagged magnetic nanoparticles (sample CR, see Table S1). In the fluorescence amplitude and phase false-color images (far right), the red and blue colors represent the emitted signal recorded under the different excitation wavelengths used. The magnetic nanoparticles were labeled with a fluorophore (Ex. = 578 nm, Em. = 613 nm, shown as red), and the nuclei (shown as blue) were imaged using the fluorescent nucleic acid stain DAPI (4′,6-diamidino-2-phenylindole dihydrochloride, Ex. = 358 nm, Em. = 461 nm).

The microscope was designed to operate as essentially two combined microscopes using multiple wavelength laser sources: an optical imaging system to measure bright field (transmission), reflection, and fluorescence images; as well as an integrated magneto-optical detection system. In all cases, the image was obtained by scanning a focused laser spot across the sample area, with the sample contained within the electromagnet. The principal components of the microscope system, together with representative images of the different optical imaging modes are shown in Figure 1 and discussed in more detail in the Methods.

In fluorescence imaging mode, the lasers were modulated at high-frequency (10 MHz) and the corresponding modulated fluorescence signal was fed through a lock-in amplifier to determine the amplitude and phase of this high-frequency fluorescence signal. From this, it was possible to obtain images corresponding to the (uncalibrated) amplitude and phase of the modulated fluorescence signal (subsequently referred to as fluorescence amplitude and fluorescence phase images). Individual lasers from the laser module unit with wavelengths suitably matched for each fluorophore excitation energy (Ex.) were used, and the filtered emission signal (Em.) recorded (Figure 1). For the example images shown in Figure 1, a nuclear membrane permeable dye was used to stain the cell nuclei (excitation = 358 nm, emission = 461, shown as false-color blue), and fluorophores bound to the MNPs were used to image the MNP distribution (excitation = 578 nm, emission = 613 nm, shown as false-color red).

The fluorescence *amplitude* image shown in Figure 1 is equivalent to fluorescence images obtained using a conventional fluorescence microscope and is useful for determining the concentration distribution of the fluorophores (or in this case fluorescently tagged MNPs). From Figure 1 the MNPs (red) surround the perimeter of the cell nuclei (blue). This perinuclear localization is a common effect seen when MNPs are internalized by cells via endocytosis.^41−43^ In contrast, the phase of the modulated fluorescence signal is related to the fluorescence lifetime of the fluorophore. The fluorescence phase image is therefore relatively insensitive to the concentration of the fluorophore, as it simply detects the change in phase of the high-frequency modulated signal when a fluorophore is present (with this phase signal scaling with the fluorescence lifetime of the fluorophore). Thus, the fluorescence phase image is equally sensitive to both the MNPs densely surrounding the nuclei and those more diffusely distributed. This effect can be seen when comparing the amplitude and phase fluorescence images in Figure 1. The latter image detects MNPs diffusely scattered over the cell membrane, which appear pink in the nuclear regions due to the superposition of the red and blue color channels. A further advantage of measuring the fluorescence phase signal is that changes in the fluorescence lifetime of the fluorophore can potentially be used to probe local environmental properties such as temperature and viscosity.^44−46^

### Probing MNPs in Suspension

The performance of the magneto-optical part of the microscope was initially evaluated by measuring aqueous suspensions of three different polydisperse MNP samples (for sample details, see Table S1, Supporting Information). For these measurements the laser spot position was fixed rather than scanned across the sample. The magneto-optical response of each magnetized sample was determined from the rotation of linearly polarized light from the sample, after the two orthogonal components were split by passing through a polarizing beam splitter. We have previously demonstrated that this magneto-optical approach can be used to measure AC hysteresis loops from MNPs in suspension, that are consistent with those measured using inductive coil-based AC magnetometry.^47^ Here, we probe both the AC susceptibility (ACS) and AC hysteresis responses using the magneto-optical method.

**Figure 2:**
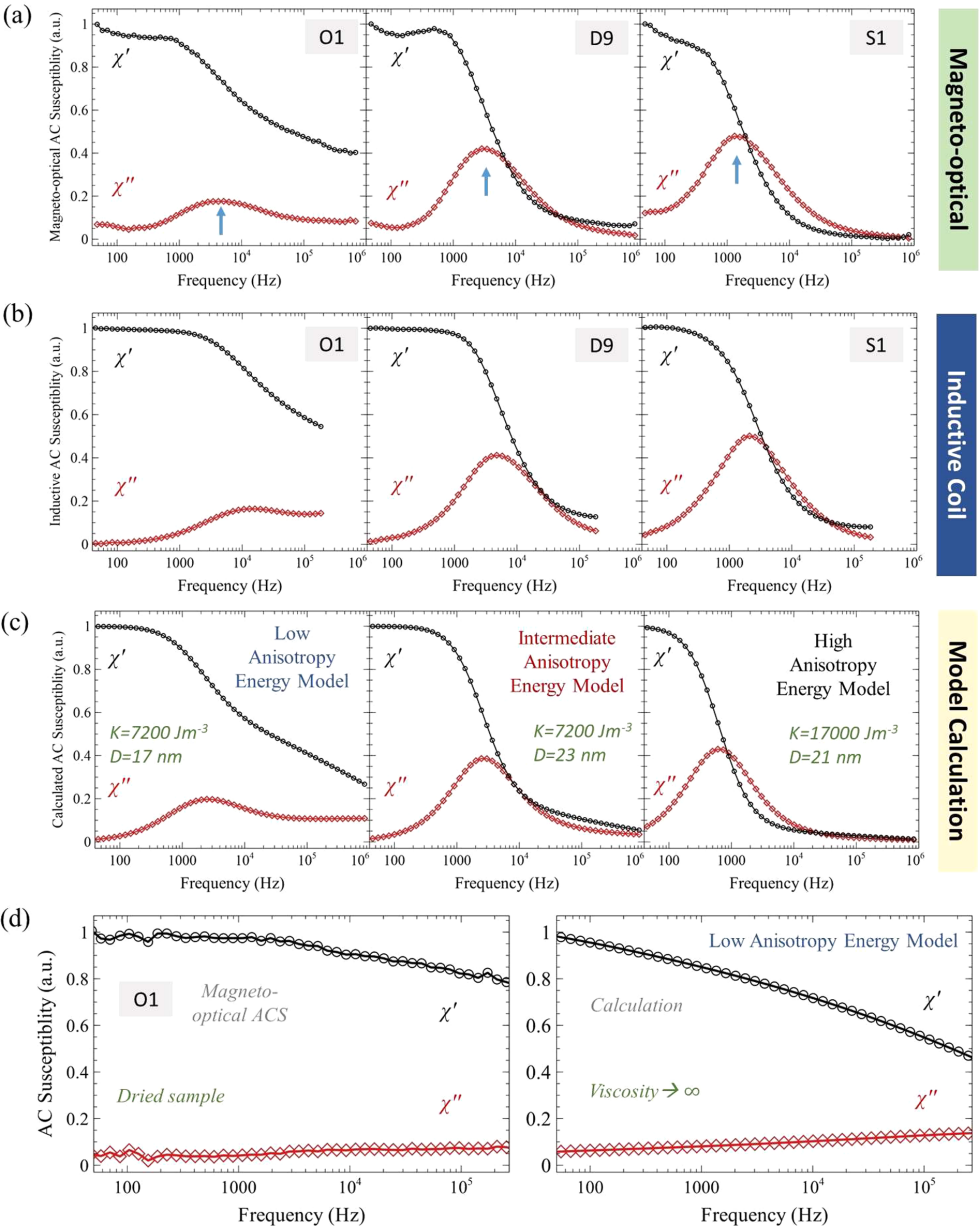
AC susceptibility (ACS) from magnetic nanoparticles in suspension. ACS measured as a function of frequency for aqueous MNP sample suspensions of O1, D9, and S1, using the magneto-optical microscope (a) and inductive coil susceptometer (b). The blue arrows in (a) indicate the position of a peak in the out-of-phase (χ′′) component of the complex AC susceptibility, caused by Brownian relaxation of the MNP clusters. Calculations performed using a model that includes MNP particle and cluster size distributions^50^ are shown in (c). The input parameters for this model were the mean MNP diameter (*D*) and polydispersity index (PDI) for each sample (values given in Figure S1), and the mean cluster size and PDI determined from DLS measurements (Table S1), with viscosity set to equal that of water (η = 7 × 10^–4^ Pas). The estimated effective anisotropy constant, *K*, was determined by qualitative fitting of the sample series measured by magneto-optical ACS, using as a starting point our previous measurements for similar sized magnetite nanoparticles,^50^ and literature values reported for maghemite nanoparticles.^52^ Part (d) shows measured magneto-optical ACS versus frequency for sample O1 dried onto a thin section of onion, and the corresponding calculation using the parameters for the low anisotropy energy model shown in (c) but with the viscosity set to represent immobilization of the particles (η = 10^8^ Pas).

The ACS as a function of field frequency, determined from the magneto-optical response, is shown in Figure 2 (a). Conventionally, ACS is measured using inductive coil-based systems, and can assess important AC magnetic properties of MNPs, as well as determining their hydrodynamic sizes and the viscosity of the surrounding suspension medium if this is not already known.^35,48−50^ The magneto-optical ACS recorded here, was found to be very similar to that measured using an inductive coil system (Figure 2 (a,b)) except for the addition of a feature at ∼ <100 Hz, caused by the effect of the motion of aggregated nanoparticle clusters on the optical signal as described in our previous study.^51^ Aside from this purely optical feature, the shape of ACS curves is dependent on a magnetic property of the MNPs known as their anisotropy energy, which determines how easily the magnetization of a particle can be realigned by a magnetic field. Nanoparticles with high anisotropy energy will require strong fields to realign their magnetization and are typically described as being magnetically “hard”. Such materials consequently display broad, open hysteresis loops. In contrast, low anisotropy energy particles, described as magnetically “soft”, require much weaker fields and consequently show much narrower (or closed) hysteresis loops.

MNP suspensions typically comprise of small clusters of stably suspended polydisperse particles (see, for example, ref (50)). For the suspensions used in this study, the distribution of cluster sizes was determined using dynamic light scattering (DLS), with measured values given in Table S1 (Supporting Information). In terms of their ACS response, clusters containing particles with sufficiently high anisotropy energies can contribute to a process known as Brownian relaxation. Here, the magnetization direction of the sample can be scattered away from the original field direction (i.e., it “relaxes”) due to the physical rotation of the clusters by Brownian motion. When the AC field frequency approaches the rate at which clusters are reoriented by Brownian relaxation (typically several kHz), a phase lag occurs between the magnetization of the cluster and the applied field direction. This is seen as a peak in the out-of-phase (χ′′) component of the ACS curve (as indicated by the blue arrows in Figure 2a). The position of this peak depends on the average cluster size in the suspension, which can tend to vary slightly with the concentration and age of the sample. In addition to Brownian relaxation, it is also possible for the internal magnetization of particles to relax by a process known as Néel relaxation. For particles with sufficiently low anisotropy energies, Néel relaxation will be a faster process than Brownian relaxation, leading to the persistence of the complex susceptibility signal up to the highest frequencies measured.

The magnetic anisotropy energy of a MNP depends on the product of the particle volume and a material dependent parameter known as the magnetic anisotropy constant (*K*). A low anisotropy energy can therefore be achieved if either the particle size and/or the anisotropy constant is small. Polydispersity in the particle size (as revealed for the samples here in the TEM images in Figure S1) will consequently lead to a distribution of anisotropy energies, and the ACS will be a superposition of signals from particles relaxing by either Brownian or Néel mechanisms. Samples that are dominated by Brownian relaxation (i.e., those containing predominately high anisotropy energy MNPs) will show a strong peak in the out-of-phase (χ′′) component of the ACS curve, with little or no susceptibility signal measured at high frequencies, whereas samples dominated by Néel relaxation (containing predominately low anisotropy energy MNPs) will show the opposite behavior.

Calculations using a model with the measured particle and cluster size distributions as input parameters, and the anisotropy constant as an output variable^50^ are shown in Figure 2c. A good, qualitative reproduction of the main features of the measured ACS curves was obtained with anisotropy constants commensurate with the MNP material for each sample (maghemite or magnetite). Brownian relaxation of clusters is suppressed when they become immobile, and so material from sample suspension O1, which ACS measurements indicated as having the greatest proportion of low anisotropy energy MNPs, were dried within a thin section cut from an onion. This onion section provided a simple but effective matrix for supporting the MNPs while allowing optical transmission. The measured magneto-optical ACS curves for this dried sample are compared to a model calculation assuming immobile MNPs, in Figure 2d, illustrating (as predicted) the persistence of the susceptibility signal due to Néel relaxation in the dried sample. These initial experiments demonstrate that magneto-optical ACS can be used to study important AC magnetic properties of the MNPs, such as their effective anisotropy, as well as to detect local physical parameters such as viscosity and particle (or cluster) sizes.

**Figure 3:**
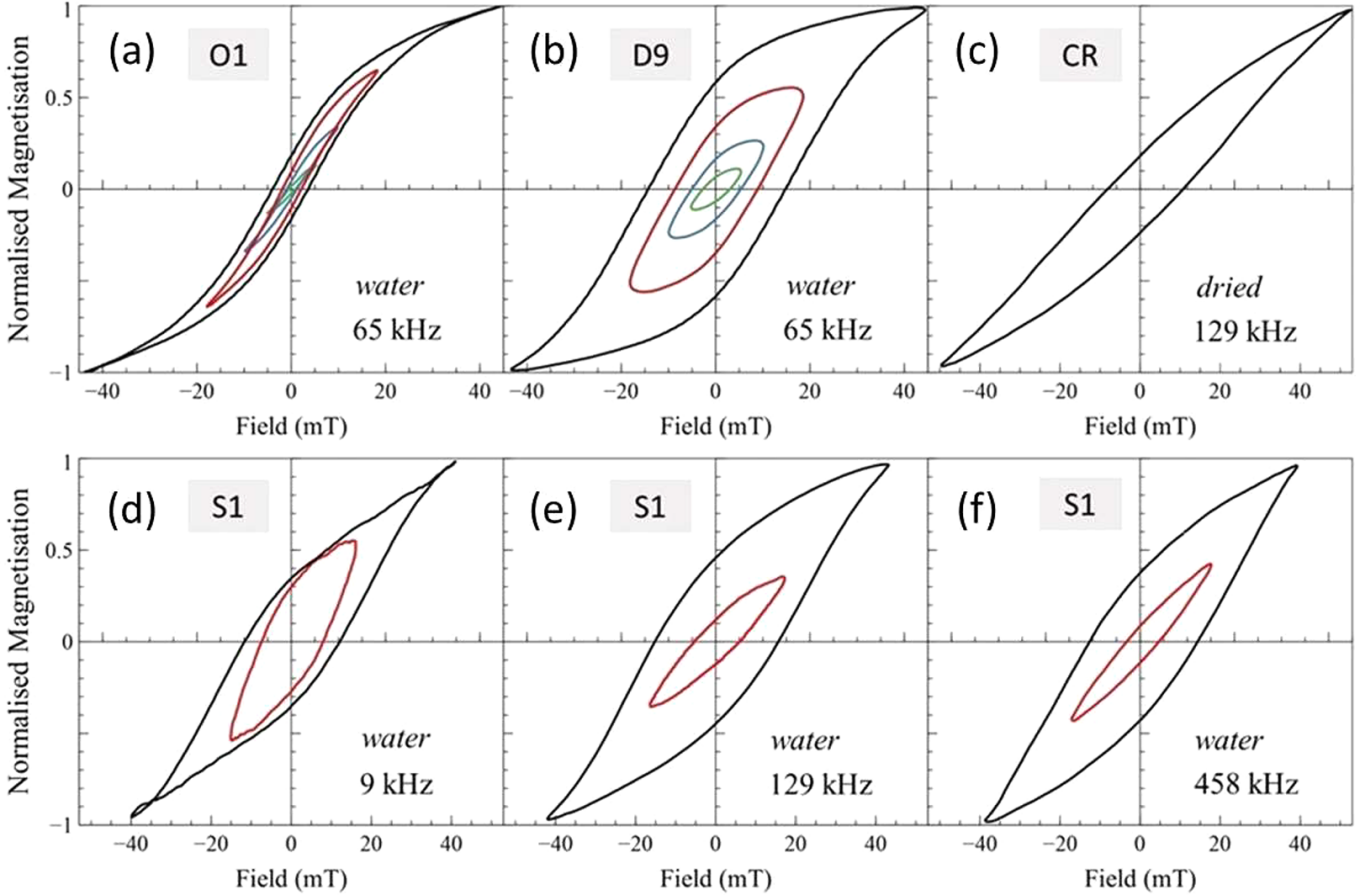
AC hysteresis loops from magnetic nanoparticles in suspension. The AC hysteresis loops from different magnetic nanoparticle samples dispersed in water were recorded at the frequencies indicated, with full-field loops shown as solid black lines and minor loops (at reduced fields) shown in other colors. The hysteresis loop obtained from a fluorescently tagged MNP sample (CR, part (c)) was measured with the sample in an immobilized state by drying it onto a thin section of onion.

The corresponding AC hysteresis loops measured at 65 kHz from the low and intermediate anisotropy energy samples (O1 and D9 respectively) are shown in Figure 3 (a,b). The shape of these loops is consistent with the anisotropy energies of the MNPs previously assessed by ACS, with sample O1 (Figure 3 (a)) showing a typical soft magnetic behavior and sample D9 (Figure 3 (b)) reminiscent of a magnetically harder material. In both cases, the sample magnetization begins to plateau at full applied field (suggesting near-saturation of the sample), while weaker fields produce characteristic unsaturated (so-called minor) hysteresis loops.

The ACS analysis of sample S1 (Figure 2 (a–c)) indicated that the anisotropy energy of the nanoparticles in this sample was high enough that mainly Brownian relaxation of the clusters would occur in the low frequency (∼kHz) range. The corresponding AC hysteresis loop measured at 9 kHz (Figure 3 (d)) reveals the behavior of the nanoparticles at a frequency slightly above the peak in the Brownian relaxation response seen in Figure 2 (sample S1, blue arrow). The shape of the loop is therefore strongly influenced by Brownian relaxation, with high induced magnetization observed even at low applied fields (as for example in the minor loop). At frequencies >100 kHz the time scales are such that Brownian relaxation is negligible. Here, subtle differences can be seen between AC loops measured at 129 kHz (Figure 3 (e)) and 458 kHz (Figure 3 (d)). A larger coercivity and more obvious plateauing of the magnetization at maximum field occurs at 129 kHz, whereas at 458 kHz the coercivity decreases slightly, and it appears the saturation field becomes higher than the maximum applied field. This is consistent with the sample becoming magnetically harder as a larger proportion of nanoparticles in the sample become blocked with increasing frequency.

### Confirmation of the Magnetic Response of MNPs Associated with Cells

The initial experiments described above demonstrate the capabilities of the magneto-optical system to measure the high-frequency AC magnetic properties of MNPs in suspension. To assess its suitability for cellular measurements, we incubated fluorescently tagged MNPs (sample CR) with cells from an osteosarcoma cell line (MG63), grown on standard glass coverslips. Prior to cellular incubation, we assessed the AC magnetic properties of the fluorescently tagged MNPs (sample CR). These were not sufficiently stable in suspension form, and so instead the AC loop obtained from a sample dried in a thin onion section was measured (Figure 3 (c)). From the particle size distribution obtained from this sample (Figure S1d), MNPs with anisotropy energies intermediate between samples O1 and S1 would be expected, as the sample consists of small particles but with a large anisotropy constant. This is qualitatively reflected in the measured hysteresis loop that while being relatively narrow does not appear to saturate at the maximum field applied.

The cells incubated with sample CR were subsequently fixed, and the coverslips mounted onto specialist glass slides with optical properties chosen to ensure a minimal contribution to the magneto-optical (Faraday) signal. The coverslips themselves present a weak magneto-optical signal due to their diamagnetic properties, which was useful for confirming the strong magnetic response from MNPs, as well as to provide a low constant background signal for AC susceptibility mapping.

**Figure 4:**
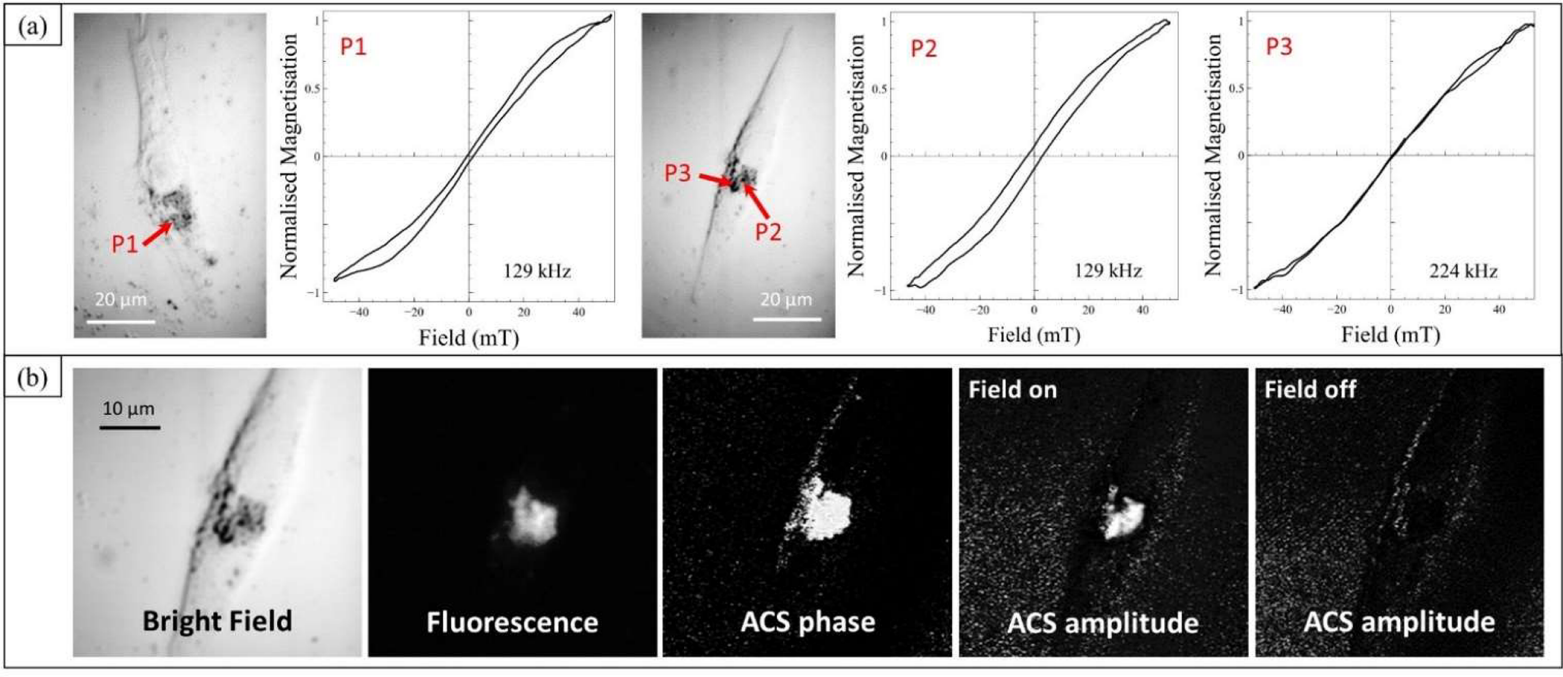
Demonstration of magneto-optical investigations on fixed cells. (a) Bright field microscopy images from two different cells found within a population of fixed osteosarcoma cells (MG63s) following incubation with fluorescently tagged MNPs (sample CR), showing point source AC hysteresis loops recorded from MNPs at different positions in the cells (labeled P1–P3), and at two different frequencies. (b) Enlargement of the perinuclear region of the right-hand cell shown in (a), measured using bright field, fluorescence (amplitude), and ACS (phase and amplitude) magnetic imaging modes. For all ACS images, the field frequency was set to 129 kHz and the amplitude was 22 mT. An ACS amplitude image is also shown measured with the field switched off, to confirm the magnetic origin of the signal from the perinuclear region of the cell.

Bright field microscopy images of the fixed cells following incubation with the fluorescently tagged MNPs (sample CR), are shown in Figure 4. AC hysteresis loops measured from different point source positions on two individual cells associated with MNPs (Figure 4 (a)) appear to show a softening of the magnetization compared to the loop obtained from the dried sample (Figure 3 (c)), with the effect being more pronounced at the higher frequency applied (224 kHz). The higher frequency measurement is reminiscent of a superparamagnetic response seen for particles with very low anisotropy energies (for examples, see the literature^24,25,35^). The differences in the loops recorded from the MNPs taken up by cells compared to the dried particles, is consistent with a change in their AC magnetization that occurs when the MNPs are densely packed into aggregated forms, enabling strong dipolar interparticle interactions to occur.^25,28,30−33,53,54^ Computational studies such as that presented by Tan et al.^55^ showed that these interparticle interactions can lead to dipolar fields that are either parallel or antiparallel to the local external magnetic field, dependent on the packing density of the aggregate. Whether this ultimately increases or decreases the AC hysteresis area (and consequently heating response) depends on how the applied magnetic field compares with the modified coercivity and saturation field of the aggregated MNPs. Interestingly, the loop measured at position P1 (Figure 4 (a)) resembles a characteristic “butterfly like” hysteresis loop that is seen when antiferromagnetic interparticle interactions dominate (see, e.g., Anand^39^).

The apparent further reduction in effective anisotropy seen between the loops measured at 129 kHz and that recorded at 224 kHz is unexpected. However, dipolar interparticle interactions are a magnetostatic effect and thus require that, during the field cycle, the magnetization of the MNPs is blocked (i.e., that the MNPs do not relax). Given the polydisperse nature of the size (and hence anisotropy energies) of the particles, aggregates will include a proportion of small particles with low anisotropy energies. Néel relaxation will occur rapidly for these particles and will be avoided only during short field cycles (i.e., at high AC field frequencies). Thus, it might be expected that dipolar interparticle interactions in such polydisperse aggregates are more prevalent at higher frequencies, consequently reducing the effective anisotropy of the aggregated particles to the point that their magnetic response tends toward superparamagnetism.

In addition to measuring these point source AC loops, it was also possible to probe the magneto-optical ACS signal while scanning over the sample area. Magnetic imaging of the cells could thus be obtained by spatially mapping the measured amplitude and phase components of the ACS signal. Owing to the weakness of the magnetic response, it was necessary to apply magnetic fields beyond the liner response region of the MNPs, and thus the amplitude and phase measured at each pixel cannot be directly related to the real and imaginary components of the ACS shown in Figure 2. However, these images reveal the spatial distribution of the MNPs, with dense clusters of MNPs observed close to the cell nucleus (as shown in Figure 4 (b)) that correlate well with the distribution of the MNPs observed in the corresponding fluorescence images. Additionally, it was found that the MNPs appeared to vanish from the ACS amplitude images when the AC field was switched off (Figure 4 (b)), confirming the magnetic sensitivity of these measurements. For the ACS phase images, contrast is provided by the phase difference between the diamagnetic glass coverslip (negative susceptibility) and the MNPs (positive susceptibility). As was the case described earlier for the fluorescence phase image, the ACS phase image is also insensitive to the amplitude of the susceptibility signal and probes only the distribution of the MNPs via their ACS phase signal. This can be seen more clearly in the higher magnification images and line-scans of this region, shown in Figure S2, where individual pixels in the ACS phase maps can be regarded as being in an “on” or “off” state depending on whether the threshold signal detection level is reached.

A different area of the same cell sample was investigated following additional wetting and remounting of the coverslip on the glass slide (Figure S3). Both low and high magnification images are presented in the figure, again showing the good correlation between fluorescence images and ACS magnetic images, particularly for the ACS phase images. The hysteresis loops measured at points P5 and P6 in Figure S3 are wider than the loops measured in the earlier cells. This suggests that the magnetization dynamics of the MNPs can vary between different cells or cellular compartments and, consequently, that the heating performance of the MNPs will show variability across a cell population.

In addition to positions P5 and P6, measurements were also performed at a point where only the glass coverslip was observed. The magnetic response from this region is typical of weak diamagnetism expected for this thin glass coverslip, confirming the high sensitivity of the magneto-optical detection. An ambiguous extracellular dark spot that could be one of many MNP clusters that appeared to have deposited directly onto the glass was also probed (yellow arrowheads in Figure S3). The magnetic signal here was also clearly dominated by the diamagnetic glass signal, weakened by the reduced optical transmission. In fact, close inspection of the position of the dark spot confirms that it does not appear in either the fluorescence or ACS maps and can thus be concluded not to contain MNPs. These results demonstrate the complementarity of the spatial sensitivity of the microscope in all imaging modes.

### Investigating the Interaction of MNPs with Cells Using Correlative Magnetic and Fluorescence Probes

The experiments above confirm the proof of concept of the magneto-optical method using MNPs that were labeled with a fluorophore. We will now show how magnetic imaging and characterization of any nanoscale magnetic material, without the requirement for added fluorescence functionality, can be combined with biological fluorescence biological imaging. In this case, we will use the combined modality to probe the magnetization dynamics of nanoparticles associated with cells. For these experiments we used HeLa cells (an immortalized cell line derived from cervical cancer cells) that were plated into channels in a commercially available glass holder designed for cell microscopy studies (see Methods section).

We will first demonstrate that magneto-optical biological microscopy can be used for magnetic imaging and analysis of live cell samples. To do this, HeLa cells were incubated with the high anisotropy energy S1 MNPs discussed above and in Figures 2 and 3. Shortly before mounting them in the microscope, the cells were stained with the cell-permeant dye calcein acetoxymethyl (AM). This dye is converted to fluorescent green calcein dye when it is transported into live cells. These first experiments were performed under ambient conditions (room temperature and pressure).

**Figure 5:**
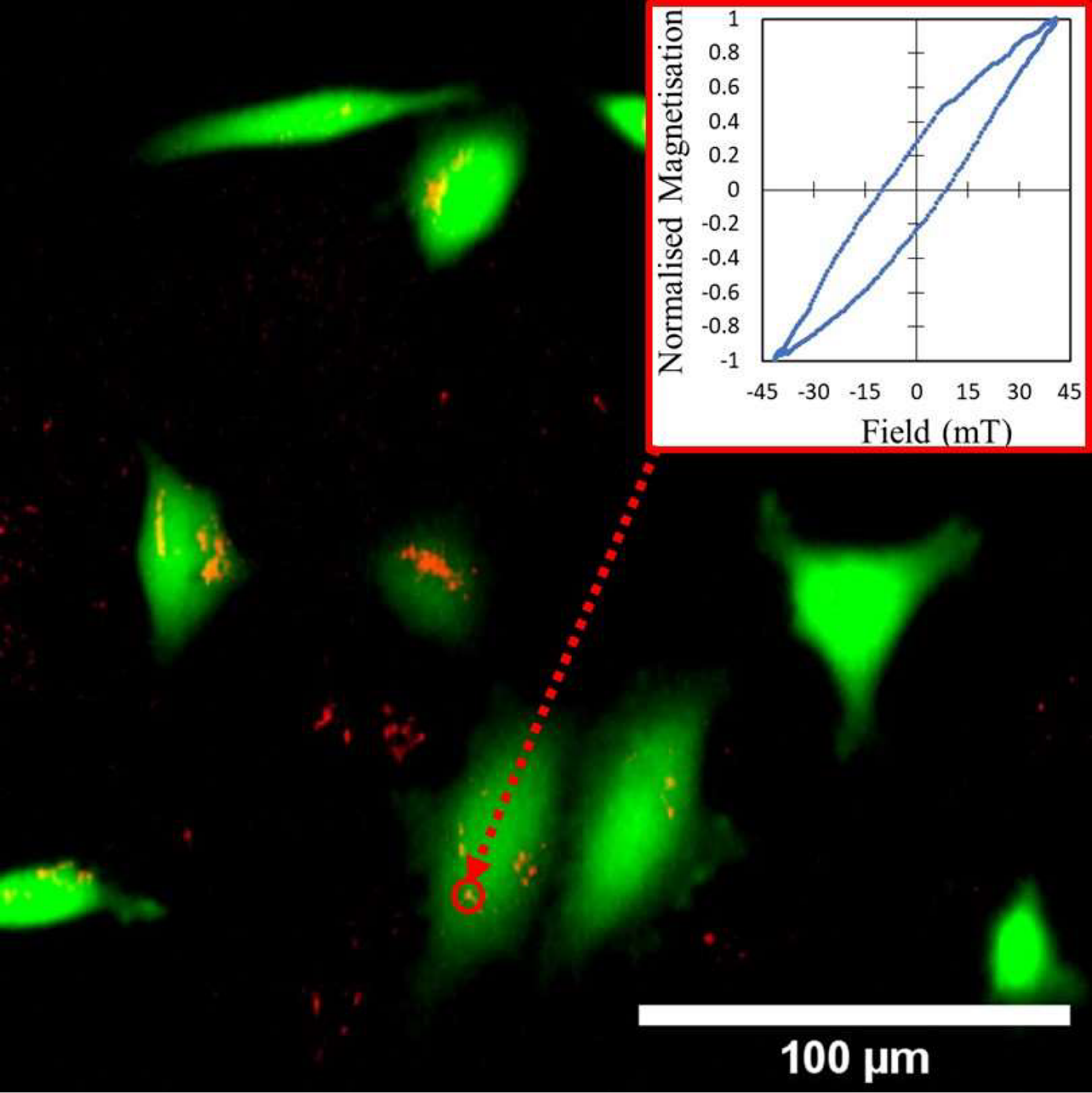
Correlative fluorescence and magnetic imaging from live cells. HeLa cells were labeled with calcein AM dye so that live cells were observed in the green fluorescence channel. Nonfluorescent MNPs (sample S1) were magnetically imaged by mapping the magneto-optical ACS amplitude signal (false color—red channel). The magnetic image was obtained at an AC field frequency of 65 kHz with a field amplitude of 22 mT. The inset shows an example point source AC hysteresis loop obtained from an aggregate of the S1MNPs measured at 65 kHz AC field frequency.

Figure 5 shows a fluorescence amplitude image recorded at the emission wavelength of the calcein dye (green), merged with the corresponding ACS amplitude magnetic image from the same region (false color, red). Many live cells can be seen in this low magnification image, displaying a typical morphology for this cell line (see, e.g., Shanmugapriya et al.^56^). Where S1 MNPs are colocalized with live cells, they appear orange/yellow in the image (due to the overlapping color channels), whereas MNPs that have deposited onto the bottom of the cell culture holder appear red. The AC hysteresis loop obtained from aggregated MNPs associated with one of the live cells (shown in the inset) again reveals a reduced AC magnetic coercivity compared to the loops obtained at various AC frequencies for the suspended form (Figure 3 (d–f)).

To investigate the origin of the altered AC magnetic behavior of the MNPs associated with cells, we attempted to correlate our analysis of the magnetization dynamics of the MNPs, with the location of intracellular vesicles known as lysosomes: membrane bound organelles containing digestive enzymes.^57^ When cells are incubated with MNPs, the particles are frequently taken into the cells by a process known as endocytosis.^58^ The end stage of this process results in the particles being sequestered into lysosomes. The combination of digestive enzymes and the low pH environment of the lysosomes can result in the dense packing of the particles, as well as their gradual degradation over time.^59^ Owing to this, it has been postulated that dense aggregation within lysosomes could be responsible for the observed changes in the magnetization dynamics of MNPs when they associate with cells.^25,30,60^

**Figure 6:**
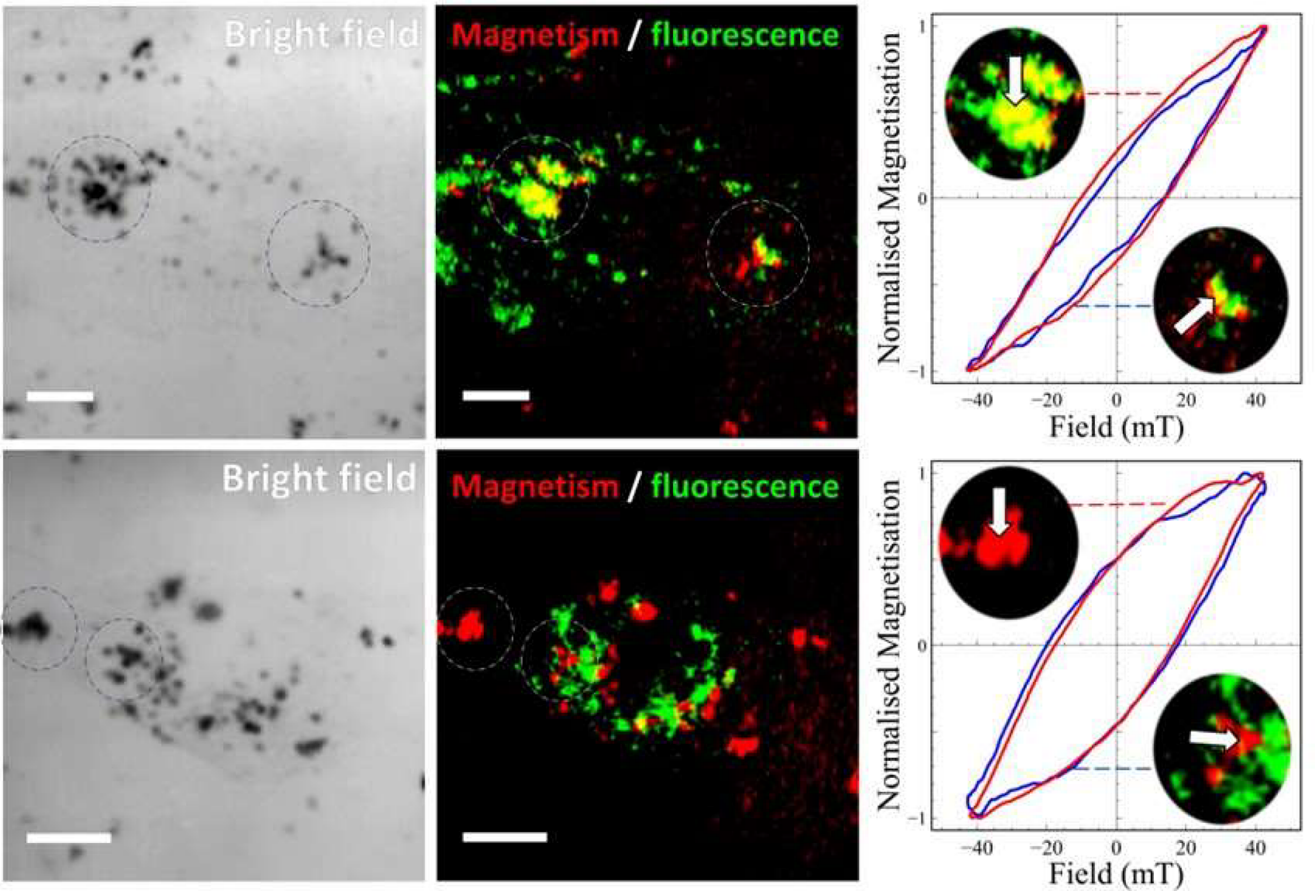
Correlative fluorescence and magnetic imaging from fixed cells with fluorescently labeled lysosomes. The lysosomes within fixed HeLa cells were labeled with green fluorescent protein and are shown in the fluorescence image (green color channel, middle images). Nonfluorescent MNPs (sample S1) were magnetically imaged by mapping the magneto-optical ACS amplitude signal (false color—red channel, middle images). The magnetic image was obtained at an AC field frequency of 65 kHz with a field amplitude of 22 mT. Colocalization of MNP aggregates with lysosomes can be seen as yellow regions (addition of the green and red color channels). Two cells were chosen for analysis that showed either a high or low proportion of colocalization (top and bottom cell images respectively). Corresponding bright field images are shown on the far left. RHS shows enlargement of two cellular regions (dashed circles in the bright field and middle images), together with point source AC hysteresis loops (white arrows) obtained from regions of colocalization (top) or no apparent colocalization (bottom). Scale bar: 10 μm.

We examined another sample of HeLa cells that were incubated with the S1 MNPs. In this case, however, the cells were transiently transfected with a green fluorescent protein (GFP) that labeled the lysosome membranes within the cells (see Methods section for details). The cells were subsequently fixed, and magnetic imaging was performed as before. The resulting merged magnetic and fluorescent images (Figure 6) show regions where aggregated MNPs were clearly colocalized with the lysosomes (appearing as yellow regions), and other regions where they were not (where the green and red regions remain separated). Point source AC hysteresis loops recorded in these different regions reveal that reduced AC magnetic coercivity occurred only where MNPs were colocalized with lysosomes. In contrast, MNPs measured in regions away from lysosomes show AC hysteresis (bottom loops in Figure 6) very similar to that observed from the same particles measured in suspension. From these results the reduction in hysteresis loop area that occurs for MNPs associated with lysosomes, would result in a significant reduction in their heating efficiency under these magnetic field conditions, compared to the same MNPs located elsewhere in the cells. The ability to measure such effects will therefore prove important when considering strategies for targeting specific cellular locations, as well as for exploring different MNP designs and magnetic field conditions which enhance the AC hysteresis loop area.

## Conclusion

We have shown here how the magneto-optical Faraday effect can be exploited as a method to probe the AC magnetic response of MNPs associated with cells, with subcellular spatial resolution. Using this approach, we have demonstrated that the distribution of MNPs can be mapped using the ACS signal from the particles and that the AC hysteresis response can be probed at spatial length scales commensurate with intracellular vesicles. Our studies suggest an apparent change in the effective magnetic anisotropy of the MNPs that varies with cellular location, MNP type, and measurement frequency used. For some particles, we found that this could lead to a superparamagnetic response (and, consequently, suppressed magnetic hyperthermia) even at high AC field frequencies. These observations are consistent with the occurrence of interparticle magnetic coupling effects, which are most likely influenced by different forms of biologically induced aggregation of the MNPs. Further, using this magneto-optical biological microscopy method, we were able to show that MNPs that were colocalized with cellular lysosomes showed a preferential reduction in their AC coercivity compared to those that were not. In this case the biochemical environment of the lysosomes may cause the formation of dense aggregates where strong interparticle interactions are favorable.

Importantly, this magneto-optical method enables the *in situ* AC magnetic response of the MNPs to be decoupled from other physical parameters such as mechanical movement and heat transfer as well as the complex biological mechanisms that initiate cell death in magnetic hyperthermia. Current controversies concerning the role of dipolar coupling in magnetic hyperthermia, tend to focus on whether aggregation either increases or reduces the effective magnetic anisotropy of the MNPs (see, e.g., Aquino et al.^61^). However, perhaps a more pertinent question for hyperthermia applications is the affect of these interparticle interactions on the AC hysteresis behavior under specific magnetic hyperthermia field conditions. The ability to probe this behavior *in situ* should help to resolve the role of aggregation on the intracellular magnetic response in magnetic hyperthermia,^33,62^ as well as providing a means to assess more complex methodologies; for example, those that include the *in situ* redistribution of nanoparticles as a design element to optimize local heating.^37^ Further, combining frequency-domain fluorescence techniques within the same microscope creates a powerful dual physical and biological microscopy technique that is well suited to help overcome the challenges for magnetic hyperthermia to become an effective cancer therapy.

Complementary magnetic-microscopy-based techniques have previously been developed that can spatially probe magnetization dynamics in nanoscale magnetic materials. These include time-resolved scanning Kerr microscopy^63^ and magnetic resonance force microscopy.^64^ However, these methods probe much faster dynamic processes, such as confined spin waves and spin-torque transfer, that occur in the GHz regime rather than the sub-MHz magnetization reversal processes of the MNPs described here. While biological magnetic force microscopy has been used to image MNPs in both cell cultures^65,66^ and tissue samples,^67^ the depth sensitivity of this method is limited, and to date only static magnetic field measurements have been performed. Thus, existing magnetic microscopy techniques are not well suited to analyze the magnetization dynamics of biologically relevant MNPs.

In addition to important biomedical applications such as magnetic hyperthermia, probing magnetization dynamics using the Faraday effect, as described here, is likely to find applications for studying the formation, distribution, and dissolution of localized magnetic material in biological systems. Examples of such applications include assessing the toxicology of magnetic nanoparticles,^68^ as well as investigating the origin of magnetic deposits in biological samples, as found in the human brain.^4,69,70^ In principle, any change in the AC magnetic response of MNPs, for example, as caused by *in situ* binding of biomolecules to the MNP surfaces, should also be detectable using the methods discussed. Such processes are important for applications where MNPs are used as diagnostic biomarkers (see examples contained within ref (71)). The methods highlighted here would thus enable a microscopic resolution complement to developing techniques such as magnetic particle spectroscopy,^71^ as well as more established techniques such as magnetic particle imaging and MRI.

## Methods

### Microscope Setup and Components

The components of the microscope are shown schematically in Figure 1. The laser module comprised three separate lasers operating at wavelengths of 405 nm (100 mW), 488 nm (25 mW), and 639 nm (150 mW). Each laser could be operated independently with different power settings, and could be digitally modulated at frequencies up to 40 MHz when required. For the experiments presented here, the 405 and 488 nm lasers were modulated at 10 MHz for fluorescence measurements, while the magneto-optical measurements were performed using the 639 nm laser in DC mode. The power of each laser was varied as necessary depending on the optical transmission of the sample, but was usually kept below 20 mW for all measurements. The output from the laser module was linearly polarized using a Glan Thomspon polarizer after exiting the XY scanning stage that used fast galvo-mirrors to raster the beam over the sample area. The beam was focused onto the sample using a long working distance (18 mm) 50× objective lens (OLYMPUS/SLMPLN50X) via holes in the electromagnet poles, providing a laser spot size of ∼ <1 μm diameter at the sample.

#### Detection of the Magneto-optical Signal

After passing through a magnetized sample, the linear polarization axis of the transmitted beam will be rotated due to the Faraday effect. This Faraday rotation is detected by passing the beam through a polarizing beam splitter that separates the light into s-polarized and p-polarized components. Each component is separately focused onto one of the two photodiodes of the balanced photodetector, which then amplifiers the difference in the signals measured at the two photodiodes. Initially the detector system was set up without a magnetized sample, to ensure that equal intensities of s-polarized and p-polarized light were incident at the photodetector. This was done by rotating the axis of linear polarization of the transmitted beam, using the half-wave plate, until the amplified photodetector output reached a minimum.

Once the system is set up in this way, subsequent Faraday rotation from a magnetized sample can then be detected as either a positive or a negative output signal from the photodetector. When an AC magnetic field is applied to the sample, the corresponding AC magnetization of the sample leads to an alternating positive and negative voltage output from the balanced photodetector, which can be recorded on an oscilloscope. The AC susceptibility is the time derivative of the AC magnetization signal. If the sample displays a linear magnetization response with applied field, for example, as found for a paramagnetic or diamagnetic material, or from MNPs measured in the linear response region, the resulting output is simply a sinusoidal wave, and the AC susceptibility can be recovered using a lock-in amplifier. However, many materials show a nonlinear response, and the resulting output therefore diverges from a pure sine wave owing to the presence of multiple harmonics that represent the AC hysteresis response of the sample. To preserve these harmonics, the photodetector must be capable of operating up to the frequency of the highest harmonic in the signal. The time-varying magnetization signal, together with the signal monitoring the applied AC field, can subsequently be captured with a suitable digital oscilloscope. Following correction for the phase shift introduced by the instrumentation and signal processing chain (described in the Magneto-optical Scanning and Imaging Methodology section below), the data can then be replotted as magnetization vs field to obtain the AC hysteresis loop of the sample.

For the experiments described here, we were interested in measuring both linear processes such as low field AC susceptibility as well as AC magnetometry where the magnetization response could be nonlinear. For optimal performance, it was therefore necessary to use two balanced photodetectors: a high bandwidth (small sensor area) detector suitable for measuring AC hysteresis loops at frequencies up to 500 kHz (using a fixed laser spot), and a large sensor area (low bandwidth) detector for measuring AC susceptibility (ACS) scans and images, as well as lower frequency (≤65 kHz) AC hysteresis loops. To optimize the positioning of the transmitted beam to accommodate for the different sensor areas of each detector configuration, the transmitted (diverging) beam was passed through an adjustable collimator (Thorlabs, SM2F) that enabled the translation of a condenser lens in order to adjust the degree of collimation of the beam.

The AC magnetometry signal was detected using the high bandwidth (75 MHz) balanced photodetector, the output from which was bandwidth limited to 10 MHz by using a low-pass filter to suppress high-frequency noise. This signal was then fed via a low noise voltage preamplifier (200 MHz bandwidth) to a high-frequency digital oscilloscope. The flat gain response achieved across a 10 MHz bandwidth with this setup enabled the inclusion of ≥20 harmonics of the AC hysteresis loop signal measured at ≤500 kHz. This ensured that a good representation of the AC hysteresis loop shape could be obtained (i.e., that sufficient harmonics were recorded) even at the highest field frequency used in the study (458 kHz). In contrast, as the ACS signal is typically measured in the linear response region, only the fundamental frequency requires detection. This was therefore measured using the large sensor area (low bandwidth; 1 MHz) photodetector, with the same filter and voltage preamplifier, but fed to a DC-50 MHz lock-in amplifier (Zurich Instruments HF2LI) in order to separate the signal into its phase and amplitude components. In addition to the magneto-optical signal, bright field (transmission) images were obtained by directly recording the monitor output from one of the sensors in the large area balanced photodetector.

#### Configuration of Electromagnet and Sample Stage

The electromagnet used was an air-cooled, ferrite core design (Ferroxcube 3F36), suitable for operating at frequencies between 20 Hz and 1 MHz, and fields from 0 to 50 mT. The ferrite core shape was modified to allow the objective lens to be positioned to within 10 mm of the sample. The samples were either positioned onto glass slides or deposited into the vessels (tracks) of a cell culture microslide (see section Cell Culture and Labeling for further details). The microslide vessels were also used for measurement of MNP suspensions, with each vessel filled with 30 μL of liquid sample. The sample positioning and movement within the electromagnet is shown schematically in Figure S4 (Supporting Information).

The AC field was generated using a signal generator (Agilent 33220a) connected to the electromagnet via a power amplifier (Newton fourth Ltd. LPA05B). For low field (<2 mT) operation (e.g., as used for ACS versus frequency scans), the magnet was used without additional capacitors in the circuit. For high field (5–50 mT) operation, a switchable capacitor unit was developed in order to operate the magnet by tuning the LCR resonant circuit to a set of discrete frequencies between 5 and 500 kHz. The field was monitored by recording the voltage drop across a known resistance (0.1 ohms, 5W) in series with the electromagnet. The performance assessment and calibration of the magnet was achieved using commercial AC field probes: for high frequencies (between 40 and 1000 kHz) a probe supplied by Nanoscience Laboratories (Probe I) was used, whereas for frequencies <10 kHz a Hirst Magnetic instruments probe (GM08) was used.

#### Measurement of Fluorescence

For both reflection and fluorescence imaging modes, the laser was modulated at 10 MHz and the reflected or filtered fluorescence beam that was fed back through the XY scanning stage was detected using a high bandwidth (200 MHz) photodetector. The photodetector signal was separated into its phase and amplitude components using the lock-in amplifier. For fluorescence imaging mode, a set of interchangeable dichroic mirrors and optical filters, matched to the excitation wavelength of the laser and emission wavelength of the fluorophore, were employed. For the proof-of-concept experiments presented here, fluorescence images were obtained simply by using the relative intensities of the respective amplitude and phase signals. As such, neither a direct optical reference for the modulated laser nor a phase calibration of the fluorescence system was required. Owing to imperfect fluorescence filtering, a very small background reflected signal from the glass slide was also present in the signal recorded for fluorescence imaging. However, this background signal was useful for providing a phase locked signal when recording fluorescence images, such that image regions not containing excited fluorophores would appear as low intensity (i.e., “black”) rather than as a randomly varying phase signal which would add white noise to the images. For the reflection imaging mode, the dichroic mirror was replaced with a 50:50 partially reflecting mirror, and the fluorescence filters were removed.

### Magneto-optical Scanning and Imaging Methodology

Scans measuring ACS as a function of frequency were obtained by recording the amplitude and phase components of the magneto-optical signal, using the lock-in amplifier. The electromagnet voltage was set such that the field was ≤2 mT for all frequencies measured in the scan, ensuring measurements were obtained in the linear field response region. Prior to measurements on MNP samples, the phase and amplitude response of the system was calibrated using a terbium doped glass standard which shows a strong paramagnetic response. As the paramagnetic standard produces a true linear magneto-optical response across the AC field frequency range, scans obtained from MNP samples could then be normalized using data from the paramagnetic standard to obtain the (arbitrarily scaled) real and imaginary (χ′ and χ′′) components of the complex ACS vs frequency. This method accounts for changes in the phase and amplitude of the signal due to frequency dependent phase shifts in the various electronic components and instrumentation used, as well as variations in the AC field amplitude with frequency. Images were also obtained using the ACS signal, but with a larger applied field (22 mT) and at a fixed frequency of either 65 kHz or 129 kHz. As with the fluorescence images, only the relative intensities of the amplitude and phase signals were recorded.

In order to obtain AC hysteresis loops, applied fields >5 mT were used, requiring the introduction of capacitors into the magnet circuit, as well as the use of different amplifier and filter settings in the signal chain. It was therefore necessary to account for the induced phase shifts of these different electronic components and instrumentation. To do this, diamagnetic glass slides were measured under identical AC field conditions (rms amplitude and frequency) to those used to measure MNP samples. The magnetization in a diamagnetic sample is linearly proportional to field, but lags the applied sinusoidal AC field by π radians (180 deg). Thus, the artificial delay introduced by the electronics could be determined for all field conditions used, by fitting sine curves to the measured time-dependent field and magneto-optical (i.e., magnetization) response measured from the glass slides, after correcting for the diamagnetic phase shift. Subsequently recorded data from MNP samples was then corrected for this instrumental delay, prior to plotting AC hysteresis loops.

### Data Acquisition and Software

The prototype microscope was interfaced to control software developed under LabView, using a National Instruments USB-6251 Multifunction I/O DAQ Device (2 DAC and a multichannel ADC) and a BNC-2120 Terminal Block. The DACs were used to control the two galvo-mirrors which raster the laser beam, and the ADC allowed a maximum sampling rate of 1 MS/channel/s. The analog inputs of the DAQ card collected the signals for each pixel in a scan frame, either directly from the fast photodetectors (for bright field and reflection images) or from the analog output of the lock-in amplifier (for fluorescence and ACS images). The latter could be set to output either a phase or amplitude signal processed by the lock-in amplifier, which was controlled using the ZI LabOne software. To optimize the acquisition time, imaging could be performed either pixel-by-pixel, line-by-line, or frame-by-frame depending on the pixel dwell time. The LabOne software was also connected to LabView control software in order to sync the instruments and collect data. The modulation and power of each laser in the module unit were individually controlled using the supplied Vortran software.

For image acquisition, the scanning galvo-mirrors allowed low pixel-resolution scanning (typically 100 × 100 pixels) to be performed at speeds close to video frame rate when measuring a strong signal (for example the bright field images). This was useful for surveying a sample to find a suitable region of interest for further study. High resolution images were acquired with the number of pixels set such that the pixel size at least matched the optical/fluorescence resolution (< ∼1 μM), but in most cases the pixel count was 5–10 times higher than this minimum. This was done to allow subsequent pixel averaging to improve signal-to-noise in the images (although no images presented here were processed in this way). For example, the high-resolution fluorescence and magnetic images shown in Figure 4b were obtained with 40k pixels. The integration time for each pixel was varied, depending on the strength of the signal. For bright-field images this was typically 10 μs, while for fluorescence imaging it varied between 0.1 and 1 ms, and for magnetic imaging between 1 and 50 ms.

### Data Processing

For all AC hysteresis loops shown, the time-dependent field and magneto-optical (magnetization) raw signals sample-averaged by the high-frequency oscilloscope were processed as follows: (i) the instrumental delay was first removed by applying the fitting procedure described in the section Magneto-optical Scanning and Imaging Methodology above; then (ii) a processed data set was obtained from the delay corrected data (field vs time, magnetization vs time) by averaging over the number of complete field cycles recorded (typically three), followed by performing a 25-point moving averaging filter, and finally replotted as magnetization vs field.

### Preparation and Characterization of Magnetic Nanoparticle Suspensions

Aqueous magnetic nanoparticle suspensions were prepared from a variety of different starting magnetite or maghemite based nanopowders, including both commercial particles and coprecipitated lab-made particles. O1 and D9 were derived from commercial Iron(III) oxide nanopowders (Sigma-Aldrich, UK). In both cases, the MNPs were stabilized in water by coating with citric acid using the method described elsewhere.^72^ After coating, the MNPs were fractionated into the two different core size population fractions to obtain samples O1 and D9 using centrifugation cycles (3000 RCF for 20 min) and magnetic separation with permanent magnets.

S1 magnetite nanoparticles were synthesized by coprecipitation of ferrous and ferric chloride in alkaline media according to procedures previously reported.^73^ Briefly, Iron(III) chloride hexahydrate and iron(II) chloride tetrahydrate (2:1 molar ratio) were dissolved in degassed deionized water under nitrogen environment (to prevent oxidation of the iron species in aqueous environments). The solution was heated to 80 °C while stirring under nitrogen. Aqueous ammonium hydroxide was added dropwise to the mixture, and the reaction was allowed to proceed for a further one h before it was transferred to a conical flask and washed to neutral pH with distilled, deionized water via magnetic separation.

In addition, a commercial suspension of dye-conjugated magnetite nanoparticles was obtained. The excitation wavelength of this dye was 578 nm with emission at 613 nm. The concentrations of all the MNP suspensions were obtained using the Ferrozine assay^74^ to determine the iron concentration. TEM measurements were performed on MNPs deposited onto carbon coated grids using a JEOL 1230 microscope. From these micrographs, particle sizing histograms were obtained using ImageJ software and fitted with a log-normal size distribution.

Dynamic light scattering measurements were performed on aqueous particle suspensions using a Malvern ZS Zetasizer in order to characterize particle cluster sizes in suspension by measurement of mean hydrodynamic sizes (*z*-average) and corresponding polydispersity index (PDI). Table SI (Supporting Information) summarizes the MNP suspensions used.

### Inductive Coil AC Susceptibility Measurements

Conventional AC susceptibility measurements were performed using a home-built inductive coil AC susceptometer, operating at frequencies between 10 Hz and 500 kHz, and at a temperature of 37 °C. For all samples, 200 μL of suspension were measured in each case. Susceptibility values were obtained following background subtraction and calibration using a known mass of the Dy_2_O_3_ powder.

### Cell Culture and Labeling

The osteosarcoma cell line MG63 (Lonza) was expanded in T-flasks in expansion media consisting of DMEM:F12 containing l-glutamine (Corning) which was supplemented with 10% fetal bovine serum (FBS) (Labtech) and 1% antibiotics and antimycotics (Sigma). MG63 were trypsinised using 1% Trypsin in PBS (Lonza) to obtain a single cell suspension and a cell count was performed. One million cells were seeded onto round coverslips (1 cm diameter) (SLS) and allowed to attach for ≥4 h. Coverslips were placed in 6-well culture plates containing 2 mL of media (Corning). Cells were cultured overnight at 37 °C, 5% CO_2_. Cells were then tagged with MNP by pipetting 300 μL of the respective MNP suspensions directly on top of the coverslips. Cultures were then again cultured overnight at 37 °C and 5% CO_2_ to allow uptake of the MNP. To fix the cells, media was aspirated from the cultures and cells washed with PBS (Sigma). Cells were fixed using 4% PFA in PBS (Sigma) for 10 min. Cells were washed again with PBS before staining with DAPI (Sigma) for 10 min. Coverslips were then washed and stored in PBS before imaging.

HeLa cells with passage number <10 were cultured in high glucose (4.5 g/L) DMEM with sodium pyruvate, supplemented with 10% FBS, 2 mM l-glutamine, 1× MEM Nonessential Amino Acids (Gibco), 100 U/mL penicillin, and 100 mg/mL streptomycin. Cells were seeded at a density of 5,000 cells/cm^2^ in vessels of ibidi μ-Slide VI-Flat (27 μL/vessel) and sealed with parafilm. After 3 h incubation at 37 degrees with 5% CO_2_, 3 μL of lysosome dye (CellLight Lysosomes-GFP, BacMam 2.0) was added to each vessel by mixing the dye with fresh prewarmed media. Cells were initially incubated for 30 h in the dark. Dilute MNPs in fresh prewarmed media were added to final concentration of 38 μg/mL and this was used to gently replace the old media in the vessels. Subsequently the cells were incubated again for 20 h in the dark, followed by washing three times and finally fixing in 10% neutral buffered formalin for 15 min. After fixation, the vessels were washed three times with PBS before imaging.

### Live Cell Imaging

HeLa cells with passage number <10 were cultured in high glucose (4.5 g/L) DMEM with sodium pyruvate, supplemented with 10% FBS, 2 mM l-glutamine, 1× MEM Nonessential Amino Acids (Gibco), 100 U/mL penicillin, and 100 mg/mL streptomycin. Cells were seeded at a density of 5,000 cells/cm^2^ in vessels of ibidi μ-Slide VI-Flat (27 μL/vessel) and sealed at the end with parafilm. After 3 h of incubation at 37 °C with 5% CO_2_, diluted MNPs in fresh warmed media with a final concentration of 38 μg/mL gently flowed into the culture vessel to replace the old media. Cells were incubated for another 42 h. Cells were washed with PBS three times and then incubated with LIVE stain (Calcein-AM) of LIVE/DEAD Cell Imaging Kit (488/570) for 10 min.

## Supporting Information Available

TEM images showing MNPs from the aqueous suspensions dried onto carbon coated grids, and the measured particle size dispersion (Figure S1); ACS phase and amplitude maps obtained from the perinuclear region of the cell shown in Figure 4 in the main paper (Figure S2); Bright-field, fluorescence, and ACS magnetic images obtained from a different cell region on the same glass coverslip as used in Figure 4 in the main paper (Figure S3); Summary of aqueous magnetic nanoparticle suspensions showing source material, coating, iron concentration of suspensions used in magneto-optical experiments, and cluster sizes as measured by dynamic light scattering (Table S1); Schematic arrangement of AC electromagnet and sample (Figure S4) (PDF)
