# Supplementary material for: Optical Microscopy Using the Faraday Effect Reveals in Situ Magnetization Dynamics of Magnetic Nanoparticles in Biological Samples
Source: ACS Nano. 2024 Feb 5;18(7):5297–310. doi: 10.1021/acsnano.3c08955 (PMC10883041; doi:10.1021/acsnano.3c08955)
Supplement: Supplementary file 1 — nn3c08955_si_001.pdf [file nn3c08955_si_001.pdf]

Supporting Information

Optical microscopy using the Faraday effect reveals *in situ* magnetization dynamics of magnetic nanoparticles in biological samples

Maneea Eizadi Sharifabad[1], *Rémy Soucaille*[2], *Xuyiling Wang*[1], *Michael Rotherham*[1,3], *Tom Loughran*[2], *James Everett*[1], *David Cabrera*[1], *Ying Yang*[1], *Robert Hicken*[2] *and Neil Telling*[1*]

[1]School of Pharmacy and Bioengineering, Keele University, Guy Hilton Research Centre, Thornburrow Drive, Stoke-on-Trent ST4 7QB, United Kingdom

[2]Department of Physics and Astronomy, University of Exeter, Stocker Road, Exeter EX4 4QL, United Kingdom

[3]Healthcare Technologies Institute, School of Chemical Engineering, University of Birmingham, Heritage Building, Mindelsohn Way, Edgbaston, Birmingham, United Kingdom

*E-mail: n.d.telling@keele.ac.uk

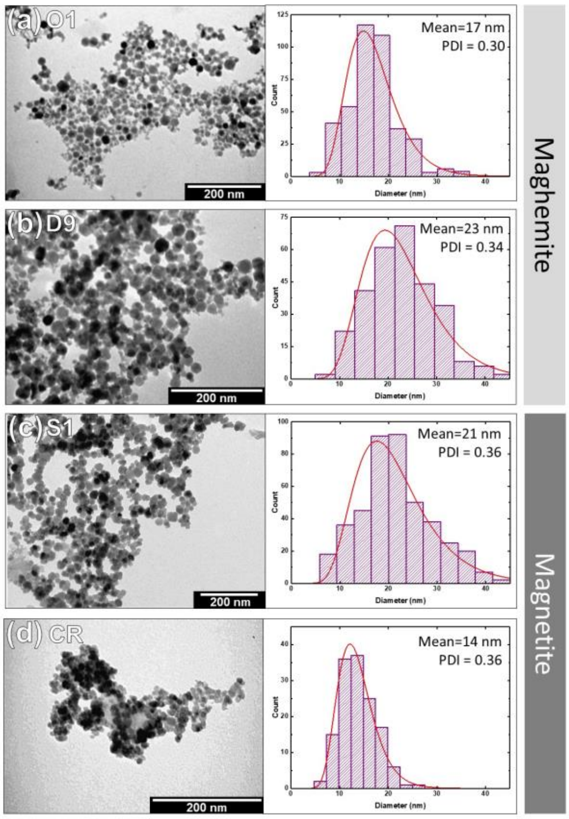

Figure S1. TEM images showing MNPs from the aqueous suspensions, dried onto carbon coated grids (LHS). RHS shows the measured (bar chart) particle size dispersion and fitted (solid line) log-normal distribution.

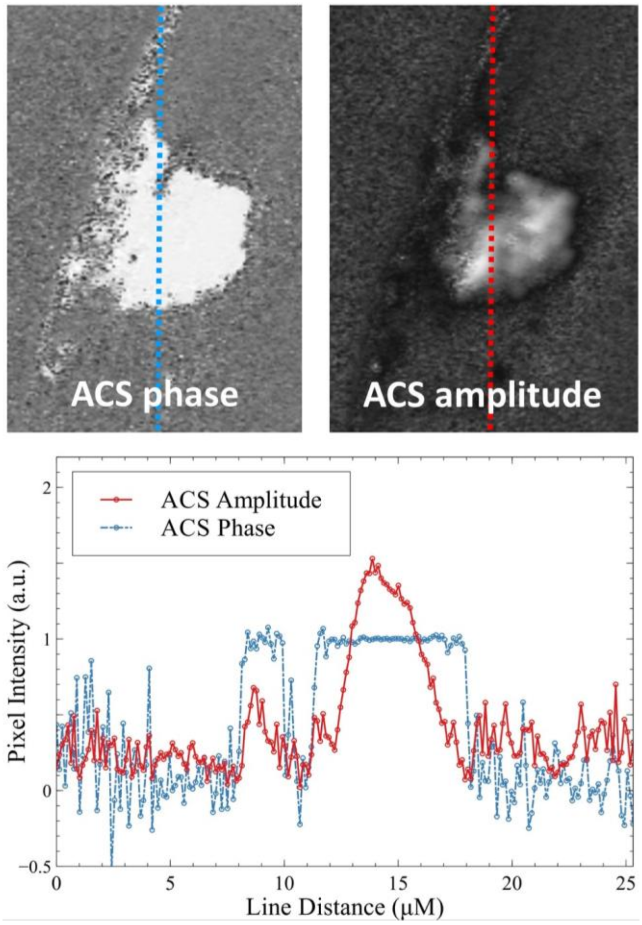

Figure S2. High magnification ACS phase and amplitude maps obtained from the perinuclear region of the cell shown in Figure 4 in the main paper (top), showing positions of the extracted line scans (dashed curves) with data from these scans plotted below. The pixel intensity shown in the lower plot represents the analogue signal from the lock-in amplifier, which was set to output either the phase or amplitude of the ACS signal. Pixel intensity values were normalized to the plateau region of the ACS phase line scan after subtracting the baseline signal recorded from regions containing only the diamagnetic glass substrate. The flat-topped line scan extracted from the ACS phase image (blue line) is in stark contrast to the varying peaks measured in the amplitude image (red line) and demonstrates the binary nature of the ACS phase signal.

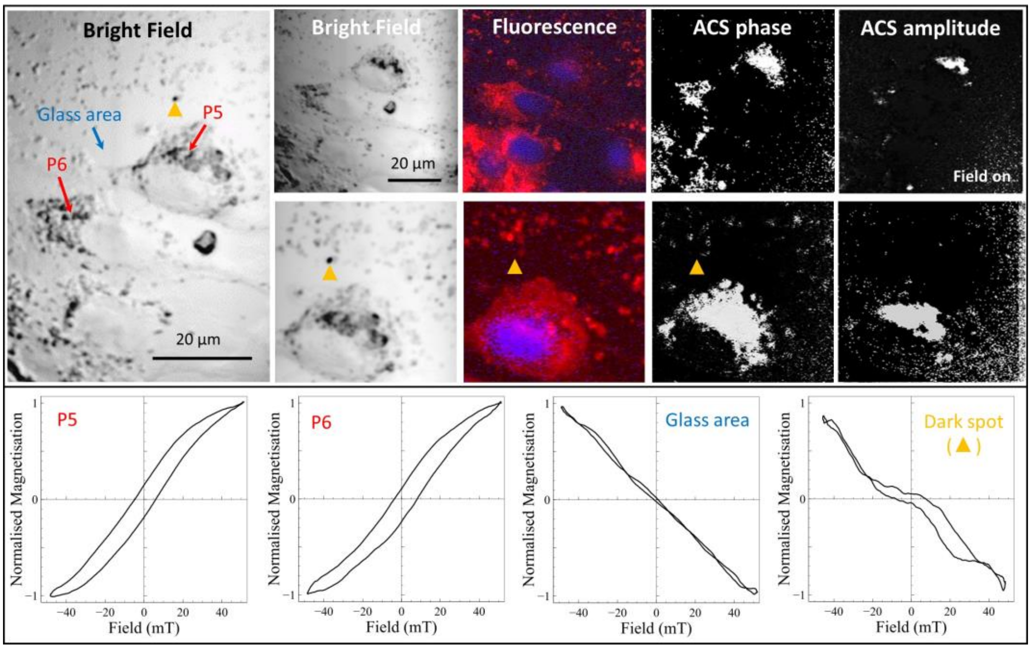

Figure S3. Identifying variable magneto-optical response from fixed cells on glass. Bright-field, fluorescence (red-MNPs, blue – cell nuclei) and ACS magnetic images obtained from a different cell region on the same glass cover slip as used in Figure 4 but measured following re-wetting and re-mounting of the sample. The lower RHS images are higher magnification images from the regions shown in the images immediately above. The lower panel shows point source AC hysteresis loops that were measured from the perinuclear region of two different cells (P5 and P6), as well as from an empty glass region, and an unidentified dark spot of material (yellow arrowhead). For all ACS images, the field frequency was set to 129 kHz and the amplitude was 22 mT.

Sample index	Material / source	Coating	Concentration (Fe µM/ml)	Particle cluster sizes	
				Z-average (nm)	PDI
O1	Maghemite nanopowder; Sigma (544884_ALDRICH)	Citric acid	11.6 ± 0.1	58	0.24
D9	Maghemite nanopowder; Sigma (544884_ALDRICH)	Citric acid	1.1 ± 0.03	59	0.13
S1	Magnetite; co-precipitated (in-house)	Citric acid	0.9 ± 0.01	91	0.15
CR	Magnetite; Chemicell GmbH (nano-screenMAG-CMX)	Carboxymethyl-dextran / fluorescent dye	3.5 ± 0.05	145	0.11

Table S1.

Summary of aqueous magnetic nanoparticle suspensions showing source material, coating, iron concentration of suspensions used in magneto-optical experiments, and cluster sizes as measured by dynamic light scattering (DLS).

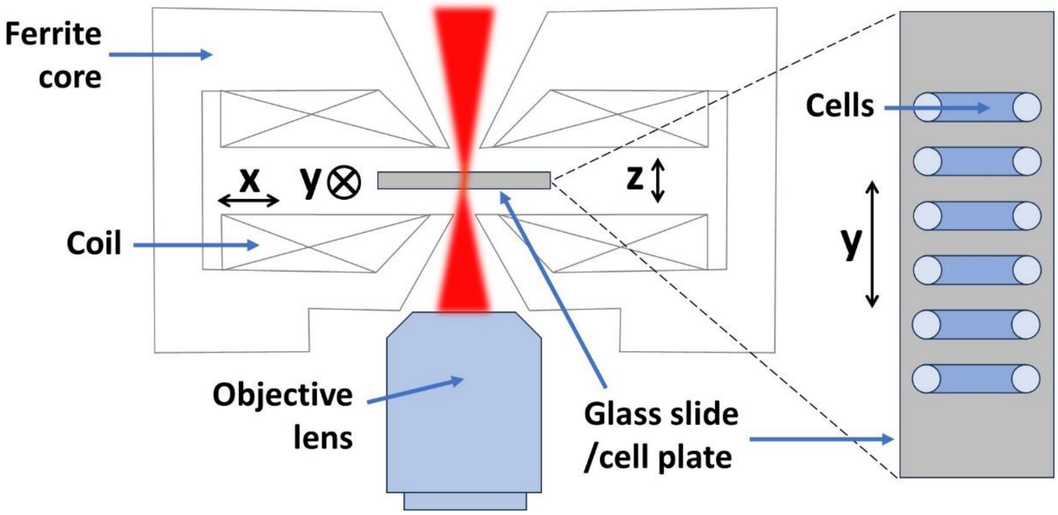

Figure S4: Schematic arrangement of AC electromagnet and sample. The sample was either deposited onto a glass slide or into vessels (tracks) within the cell culture microslide container (shown in plan-view on the right-hand side). One end of the glass slide / cell plate was fixed to a sample translation stage positioned outside of the electromagnet (not shown) that enabled movement in the x-y plane (for sample surveying) or along the z-axis (for focusing). The distance between the two coil surfaces was ~5mm and the distance from the objective lens to the central sample z-position was ~10 mm.
